# Essential roles of mitochondrial and heme function in lung cancer bioenergetics and tumorigenesis

**DOI:** 10.1186/s13578-018-0257-8

**Published:** 2018-11-02

**Authors:** Sarada Preeta Kalainayakan, Keely E. FitzGerald, Purna Chaitanya Konduri, Chantal Vidal, Li Zhang

**Affiliations:** Department of Biological Sciences, University of Texas at Dallas, Richardson, TX USA

## Abstract

Contrary to Warburg’s hypothesis, mitochondrial oxidative phosphorylation (OXPHOS) contributes significantly to fueling cancer cells. Several recent studies have demonstrated that radiotherapy-resistant and chemotherapy-resistant cancer cells depend on OXPHOS for survival and progression. Several cancers exhibit an increased risk in association with heme intake. Mitochondria are widely known to carry out oxidative phosphorylation. In addition, mitochondria are also involved in heme synthesis. Heme serves as a prosthetic group for several proteins that constitute the complexes of mitochondrial electron transport chain. Therefore, heme plays a pivotal role in OXPHOS and oxygen consumption. Further, lung cancer cells exhibit heme accumulation and require heme for proliferation and invasion in vitro. Abnormalities in mitochondrial biogenesis and mutations are implicated in cancer. This review delves into mitochondrial OXPHOS and lesser explored area of heme metabolism in lung cancer.

## Background

**Fig. 1:**
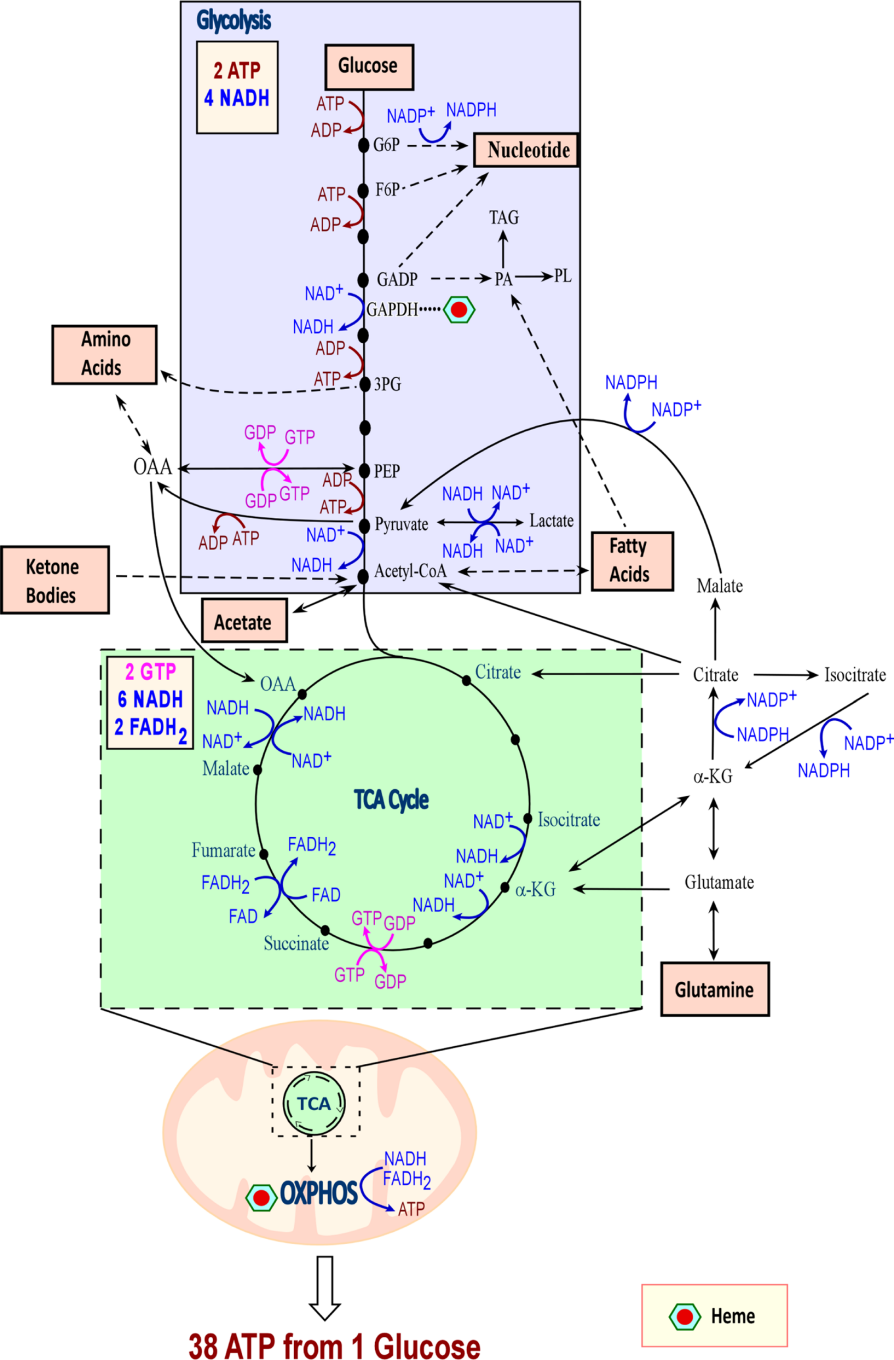
The essential bioenergetic metabolic processes. Cells generate cellular energy ATP via glycolysis and TCA cycle coupled with OXPHOS. Although cancer cells utilize a high amount of glucose, as Warburg originally observed, recent experimental data have shown that glucose is oxidized in lung tumors of NSCLC patients [4]. Additionally, cells are able to use a variety of bioenergetic substrates, including glucose, amino acids, acetate, fatty acids, glutamine, and ketone bodies to support cell growth. The numbers of ATP, GTP, NADH, FADH_2_ generated when one molecule of glucose is consumed following glycolysis, TCA cycle, and oxidative phosphorylation are also shown. Synthesis or utilization of ATP/ADP are shown in red, while NAD^+^/NADH are shown in blue, and GTP/GDP are shown in pink. *G6P* glucose-6-phosphate, *F6P* fructose-6-phosphate, *GADP* glyceraldehydes-3-phosphate, *OAA* oxaloacetate, *3PG* 3-phosphoglycerate, *PEP* phosphoenolpyruvate, *αKG* α-ketoglutarate, *OXPHOS* oxidative phosphorylation, *GAPDH* glyceraldehydes 3-phosphate dehydrogenase, *PA* phosphatidic acid, *TAG* triacylglycerol, *PL* phospholipid

At the cellular level, energetic transactions underlie all processes. There are several energetic currencies of the cell, but most of these interactions utilize adenosine triphosphate, or ATP. There are two primary pathways to synthesize this important molecule: glycolysis and oxidative phosphorylation. The term glycolysis is derived from the words “glyco,” meaning sweet, and “lysis,” meaning breaking. Therefore, it is unsurprising that glycolysis is the process of breaking down sugar to form cellular energy. The substrate for this process is usually glucose, and two ATP molecules are produced for every molecule of glucose. As this process does not require oxygen, it is alternatively referred to as anaerobic respiration. To begin glycolysis, glucose transporters facilitate glucose uptake [1]. Next, glucose is phosphorylated by hexokinase to become glucose-6-phosphate. Glucose-6-phosphate is then acted on by glucose-6-phosphate isomerase and becomes fructose-6-phosphate. These phosphorylated sugars can then enter the pentose phosphate pathway, creating nucleotides and NADPH, or the glycolytic pathway, which synthesizes lactate. Phosphofructose-1 then facilitates the transformation of fructose-6-phosphate to fructose-1,6 bisphosphate. Fructose-1,6 bisphosphate can either continue down the glycolytic pathway and become glyceraldehyde-3-phosphate or dihydroxyacetone phosphate for use with lipid synthesis (Fig. 1). Glyceraldehyde-3-phosphate is transformed into glycerate-2-phosphate by glyceraldehyde-3-phosphate dehydrogenase. Enolase then changes glycerate-2-phosphate into phosphoenolpyruvate (PEP) (Fig. 1). Pyruvate kinase then produces two molecules of ATP and two molecules of pyruvate from two molecules of PEP. Pyruvate is then converted into lactate by lactate dehydrogenase-A, converting NADH to NAD^+^. NAD^+^ is critical because it feeds the cyclic nature of the glycolysis (Fig. 1).

**Fig. 2:**
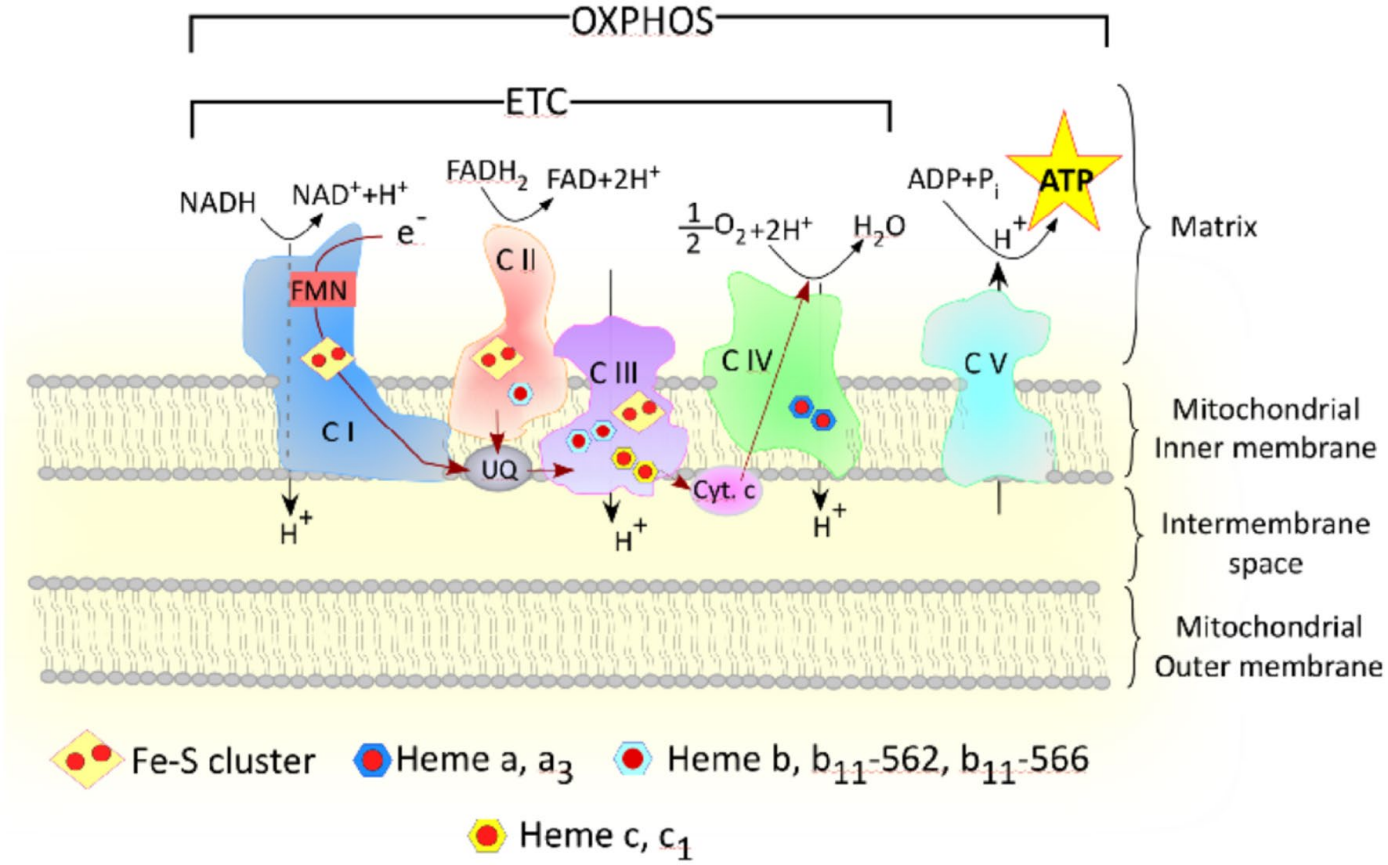
Multiple forms of heme are required for the proper functioning of mitochondrial OXPHOS Complexes. Heme serves as the prosthetic group for many mitochondrial respiratory complexes. This cartoon demonstrates the different types of heme that serve as prosthetic groups for mitochondrial complexes—complex II, complex III, and complex IV. Hence, heme serves a pivotal role in mitochondrial oxidative phosphorylation. Indicated in red is the direction of electron transport through a series of transporters embedded in the mitochondrial inner membrane that shuttles electrons from NADH and FADH_2_ to molecular oxygen

Oxidative phosphorylation (OXPHOS), also known as aerobic mitochondrial respiration, is much more complex than glycolysis. As the name suggests, oxidative phosphorylation requires oxygen. It is also slower than glycolysis. However, oxidative phosphorylation is substantially more fruitful than glycolysis. Oxidative phosphorylation is made possible by the mitochondrial respiratory chain complexes I–V. Oxidative phosphorylation begins when pyruvate is imported into mitochondria through the outer mitochondrial membrane, the intermembrane space, and the inner mitochondrial membrane by mitochondrial pyruvate carrier (MPC). Pyruvate can also be transformed into lactate and nicotinamide adenine dinucleotide (NAD) via lactate dehydrogenase (LDH). Within the mitochondrial matrix, pyruvate is changed to acetyl coenzyme A (acetyl CoA) through pyruvate dehydrogenase catalysis. Acetyl CoA combines with oxaloacetate in the first step of the tricarboxylic acid cycle (TCA cycle, Fig. 1), and through organic acid oxidation, succinate is formed. NADH then donates electrons to the electron transport chain, which includes mitochondrial complexes I–IV (Fig. 2). Mitochondrial complex I, also known as NADH dehydrogenase or NADH ubiquinone oxidoreductase, is the largest mitochondrial complex and begins the electron transport chain [2]. Electrons then shift from mitochondrial complex I to coenzyme Q. Some electrons come directly to coenzyme Q from NADH. Flavin-containing enzyme complexes mediate this transition of electrons [3] (Fig. 2). Complex II or III can both be the next destination for these electrons. Succinate becomes reduced, and these electrons come to coenzyme Q or complex III. Complex III and cytochrome c then pass on electrons to complex IV. A proton gradient is formed by complexes I, II, IV as they pass electrons over the inner mitochondrial membrane. Protons then enter the mitochondrial matrix with the help of complex V, ATP synthase, and result in the production of ATP. Complexes I–V allow oxidative phosphorylation and can produce up to 38 mol ATP per mol of glucose (Figs. 1 and 2). The importance of TCA cycle and oxidative metabolism in lung cancer is highlighted by recent studies of NSCLC tumors in human patients showing that glucose metabolites from enhanced glycolysis and lactate enter and intensify the TCA cycle, although NSCLC tumors are metabolically heterogeneous [4, 5]. A study using genetically engineered mouse models (GEMMs) for lung cancer (*Kras*^*LSL*-*G12D/*+^*Trp53*^−*/*−^ and *Kras*^*LSL*-*G12D/*+^*Lkb1*^−*/*−^) showed that the contribution of lactate to the TCA cycle is higher than that of glucose [6]. Additionally, components of OXPHOS complexes and markers of mitochondrial biogenesis are found to be highly predictive of reduced overall survival in NSCLC patients [7]. Likewise, the expression of OXPHOS genes is negatively correlated with the prognosis of lung adenocarcinoma [8].

## Prominent genetic differences exist between healthy tissue and lung cancer tissue

The value of genetic drivers in lung cancer cannot be overstated. It is believed that up to 60% of lung adenocarcinomas have driver mutations. These mutations most commonly occur in proto-oncogene B-Raf (BRAF), kirsten rat sarcoma viral oncogene (KRAS), anaplastic lymphoma kinase (ALK), and epidermal growth factor receptor (EGFR) [9]. In lung cancer, the EGFR pathway is the main signaling pathway. The mutation rate of genes in the EGFR pathway are as high as 70–80% in lung cancer tissues [9, 10]. This high rate is attributable to the fact that EGFR can act via the PI3K/AKT/mTOR, RAS/RAF/MAPK and JAK/STAT signalling pathways [11–13]. Activating mutations in *EGFR* lead to constitutive tyrosine kinase activation and oncogenic transformation of lung epithelial cells in vitro [13, 14]. Activating mutations of *EGFR* were found in 10–15% of unselected Western patients [15–18] and 30–40% of Asian patients [19–21].

KRAS mutations are responsible for around 30–35% of lung adenocarcinoma genetic variation, 97% of which occur on codon 12 or 13 [9]. Ras mutations frequently occur in human cancers [22]. Insensitivity to GTPase-activating proteins is caused by activating mutations in Ras guanosine nucleotide-binding proteins [23]. Glucose uptake and flux, important for the survival and proliferation of lung cancer, are partially promoted by activated GTP-bound Ras family members. Oxygen consumption and tricarboxylic acid cycle activity are also increased by activated Ras, further fueling the metastatic capacity of cancer cells. Ras activates cytochrome c oxidase, a vital component of complex IV of the electron transport chain containing 10 genomic DNA-encoded subunits and 3 mitochondrial DNA-encoded subunits [24, 25]. The levels of cytochrome c oxidase are elevated in some forms of cancer [25, 26]. A549, a lung adenocarcinoma cell line, is unable to grow without activated cytochrome c oxidase [23]. Ras mutations are far from the only nuclear genes with frequent mutations in lung cancer. Genes for isocitrate dehydrogenase, including IDH1 and IDH2, and succinate dehydrogenase, including SDHB, SDHC, and SDHD, code for mitochondrial components [27]. As structure determines function, the deformities caused by these mutations affect mitochondrial function. This altered mitochondrial function is either causal to or associated with lung cancer.

In addition to genomic differences, lung adenocarcinoma exhibits proteomic differences. ATP synthase subunit d (ATP5D) are expressed in much higher levels in cancerous tissues than in healthy tissues [28]. Similarly, two important enzymes associated with aerobic glycolysis and fatty acid synthesis, malic enzyme (ME) and ATP citrate lyase (ACLY), are highly associated with non-small cell lung cancer (NSCLC) cells. ME catalyzes decarboxylation of malate to yield pyruvate and CO_2_, accompanied by the production of NADH or NADPH. ACLY is a key enzyme of de novo fatty acid synthesis responsible for generating cytosolic acetyl-CoA and oxaloacetate from citrate. Akt directly phosphorylates and activates ACLY [29]. The expression levels of phospho-Akt and phospho-ACLY are positively correlated, and ACLY is directly activated by the PI3K-Akt pathway in lung adenocarcinoma cell lines [30]. Activated ACLY is a negative prognostic factor in human lung adenocarcinomas. ME correlates with metastases to mediastinal lymph nodes and ATP-citrate lyase correlates with local tumor stage. Interestingly, upregulation of these two enzymes is associated with increased survival rates in young patients and decreased survival rates in elderly patients [31]. ME levels are also increased in the lung tissues of smokers compared to non-smokers [31]. Nonsmokers, however, are believed to have adenocarcinomas that evolve locally. Non-smokers with adenocarcinoma displayed four times as many differentially expressed genes than smokers with adenocarcinoma did [32]. Lung adenocarcinomas are the most common form of NSCLCs found in non-smokers. Interestingly, non-smokers and smokers appear to follow very different modes of tumorigenesis. It is believed that in smokers with adenocarcinoma, a small area within the lung becomes genetically altered. From there, it evolves into cancer. Fascinatingly, the level of upregulation of some genes in smokers is correlated with the frequency that the smoker smoked [32].

From the enormous body of research on the genetic basis of lung cancer, it is obvious that lung cancer is highly genetically heterogeneous. Therefore, it is unsurprising that chemo-resistance is a major problem for lung cancer patients. Any resistant cells will replicate after the cancer is subjected to the first treatment, and therefore the drug will quickly lose efficacy in a heterogeneous tumor as the tumor acquires genetic and epigenetic changes [33]. Understanding both inter-tumoral and intra-tumoral heterogeneity is consequently of great value when seeking combination therapies for lung cancer.

## Elevated heme uptake and synthesis are hallmarks of NSCLC cells

Iron protoporphyrin IX, also known as heme, is linked with oxygen utilization, transport, and storage. Heme enters cells through two pathways. Most mammalian cells possess machineries allowing de novo synthesis of heme. In addition, most cells possess heme transporters, such as HCP1 (heme carrier protein 1) and HRG1 (heme related gene 1), for heme uptake. Heme serves as a prosthetic group for hemoglobin, myoglobin, catalases, peroxidases, cytochromes, and several mitochondrial respiratory complexes (Fig. 2) [34, 35]. Heme also has a plethora of biological functions, and is important for circadian rhythm, pancreatic development, neurogenesis, and erythroid biogenesis [36]. Heme directly regulates many vital cellular processes that include cell cycle, cell death, transcription, and translation [37–41]. Abnormal levels of heme are associated with diverse disease states, including anemia; porphyria; neurodegenerative disorders; type-II diabetes; coronary heart disease; and lung, pancreatic, and colorectal cancers [37, 42]. Therefore, heme levels are tightly regulated.

There have been a plethora of epidemiological studies linking dietary heme or red-meat intake with increased risks of several cancers [37, 43–46]. A meta-analysis by Gnagnarella et al. [47] showed that high consumption of red meat is associated a statistically significant 24% increased risk of lung cancer. A previous review article extensively discussed epidemiological studies linking dietary heme intake with increased risks of various cancers [37]. Thus, this article will focus mainly on the molecular and cellular actions of heme germane to lung cancer. Studies from the authors' lab implicated heme in lung cancer development [37, 42]. We have shown that NSCLC cell lines exhibited elevated levels of heme synthesis relative to the nontumorigenic cell line HBEC30KT representing normal cells. Inhibition of the rate-limiting heme synthetic enzyme 5-aminolevulic acid synthase (ALAS1) resulted in inhibition of cell proliferation and migration of NSCLC cell lines [42]. This suggests that heme is crucial for progression and metastasis of NSCLC cell lines, albeit in vitro. NSCLC cells also exhibited elevated OXPHOS and elevated expression of oxygen-utilizing hemoproteins, such as CYP1B1 (cytochrome P450 family 1 subfamily B member 1) and cytoglobin. One important function of heme is to serve as a cofactor or prosthetic group for many proteins and enzymes, particularly those involved in oxygen transport, oxygen utilization, and oxygen detoxification [36]. Elevated heme synthesis and uptake should increase cellular heme availability and intensify the synthesis of oxygen-utilizing hemoproteins to support tumorigenic functions [37]. Hemoproteins constitute the mitochondrial electron transport chain complexes that carry out mitochondrial OXPHOS for energy production [42]. Notably, one enzyme in glycolysis, GAPDH (glyceraldehyde-3-phosphate dehydrogenase), has been shown to have a function in heme delivery [48] (Fig. 1) while multiple subunits require heme (Fig. 2). These provide intrinsic links between heme and OXPHOS and cellular bioenergetics.

## Mitochondrial DNA mutations and changes in copy number are associated with lung cancer

Somatic and germline mitochondrial mutations may both be carcinogenic. However, while most DNA within the body is protected by histones and introns, mitochondrial DNA lacks these important safeguards [49]. Mitochondria also lack DNA-repair machineries present in the nucleus [50]. Therefore, mitochondrial DNA is especially prone to mutations when exposed to reactive oxygen species (ROS). The resulting instability causes both changes in copy number and mutations [51].

According to the multiple hit hypothesis, mitochondrial DNA may function as a complementary gene mutation or as a driver, allowing cancer cells to possess increased clonogenic and/or mutagenic capabilities [51]. Mitochondria increase their copy number substantially to avoid these mutations. The copy number may be changing during the epithelial-to-mesenchymal transition, which is thought to be the most important stage for metastatic potential [52]. For example, in A549 cells, copy number increased from 1700 to 2800 during the epithelial-to-mesenchymal transition. According to some studies, carcinogenicity is correlated with the copy number of mitochondrial DNA, an association drawn especially in lung adenocarcinoma [53–55]. The sets of genes associated with the tricarboxylic acid cycle and the respiratory electron transport have the strongest correlation. However, this may be skewed by diseases that cause both cancer and increased mitochondrial copy number. To ascertain whether this relationship holds true outside of latent diseased states, peripheral white blood cells were tested for increased mitochondrial DNA copy number. These studies indicated that even after correcting for latent disease, mitochondrial copy number is still associated with increased risk of lung cancer [49]. Lung adenocarcinoma is noted to exhibit increased copy number. However, this is not true for all forms of cancer, as is evidenced by some cancers that are associated with decreased copy numbers [54, 55].

Another factor affecting mitochondrial copy number is the expression of transcription factor A, mitochondria, also known as TFAM [55]. Because TFAM binds to mitochondrial DNA, it helps control mitochondrial gene expression. TFAM expression is positively correlated with mitochondrial DNA copy number [55, 56].

Genes associated with mitochondrial respiration are also differentially expressed in lung tumor cells. In lung adenocarcinoma, 1000 differentially expressed genes, including 535 upregulated genes and 465 down regulated genes, were found utilizing matched tissues from the same patient. Genes associated with the mitochondrial oxidative phosphorylation and the electron transport chain constitutes one class of differentially upregulated genes. UQCRC2, UQCR11, NDUFA1, NDUFA2, NDUFA7, NDUFB1, NDUFB8, NDUFV1, NDUFV2, NDUFS3, NDUFS7, and ATP5D are related to bioenergetic pathways, including the electron transport chain and mitochondrial ATP synthesis coupled to electron transport [9]. Genes specific to mitochondrial biogenesis are also upregulated in circulating lung cancer cells and seem to be imperative for metastatic potential. For example, peroxisome proliferator-activated receptor gamma coactivator 1 alpha, also known as PGC-1α, was found to increase oxygen consumption, mitochondrial biogenesis, and oxidative phosphorylation, thereby fueling metastases. Interestingly, LeBleu et al. [57] found that PGC-1α only seemed to affect metastases, and had no demonstrated effect on cancer cell proliferation, epithelial-to-mesenchymal transfer, or primary tumor growth.

## Inhibition of mitochondrial function inhibits lung tumor progression

Oxidative phosphorylation is crucial for anchorage-independent cancer cell proliferation [58]. Suppression of oxidative phosphorylation substantially limits the tumorigenic capacity of cancer cells. Targeting mitochondria, which are necessary for the electron transport chain and oxidative phosphorylation, starves cancer cells of ATP and limits growth and metastasis of tumors. Studies have shown intensified oxygen consumption in NSCLC cell lines HCC4017 when compared to the normal HBEC30KT cells isolated from the same patient. Hypoglycemia also correlates with higher oxygen consumption and encourage glutamine consumption in lung cancer cells. Conversely, low levels of glutamine correlate with low oxygen consumption and encourage glucose utilization [59]. Therefore, when mitochondria are targeted, lung tumor cells become more susceptible to cytotoxic drugs. Several treatments have been shown to interfere with normal mitochondrial function in lung tumor cells, including metformin, BAY87-2243, a lead structure; and microRNA-126 [60–63]. These treatments target different aspects of mitochondrial function.

Cyclopamine, a known inhibitor of Hedgehog signaling pathway, has been shown to exhibit anti-carcinogenic properties. Cyclopamine tartrate, a water-soluble analog of cyclopamine, and is therefore, a better potential therapeutic agent. Cyclopamine and cyclopamine tartrate inhibit smoothened (SMO), which facilitates Hedgehog signaling. This agent also generates ROS, which perturb tumor cell mitochondria. Cyclopamine tartrate can induce mitochondrial fission and fragmentation in some NSCLC cell lines, including A549, H1299, and H460 cells, impeding mitochondrial respiration [60].

Metformin, which has been historically used to alleviate type II diabetes, was noted in 2001 to have anticancer properties in mammals [64]. Patients taking metformin also had fewer instances of cancer than individuals who did not [65–69]. Metformin reduces oxygen consumption in the presence of pyruvate and malate, starving mitochondrial complex I of its substrate: NADH. Metformin may also disrupt the lipid metabolism, glucose metabolism, tricarboxylic acid cycle, the methionine cycle, the folate cycle, and nucleotide synthesis [64, 70–72].

Although most cells have mitochondria, not all cells are strongly affected by mitochondrial inhibitors. Healthy cells likely have lower energy requirements than tumor cells and consequently are not as strongly affected my mitochondrial inhibitors. Healthy cells can maintain functionality even in the presence of mitochondrial inhibitors, making these agents viable treatment options.

## Stromal cells in the tumor microenvironment contribute bioenergetics molecules to cancer cells

**Fig. 3:**
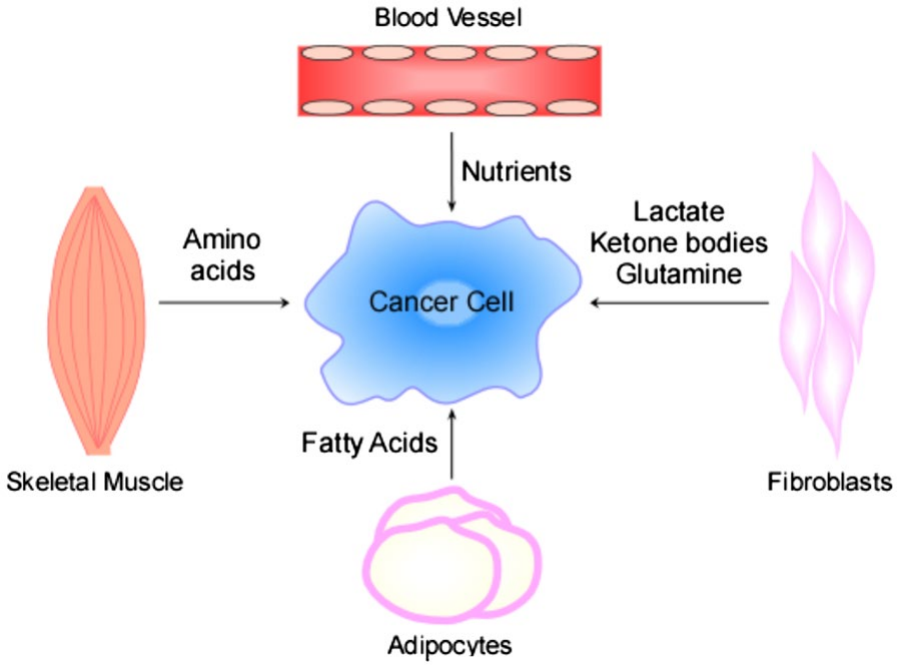
Nutrient exchange in tumor microenvironment. Tumor cells receive metabolites from the neighboring stromal cells. Blood vessels supply nutrients to the cancer cells. Fibroblasts secrete lactate and ketone bodies as a result of metabolic shift to glycolysis in response to hydrogen peroxide released by cancer cells. They undergo autophagy to secrete glutamine. Adipocytes secrete fatty acids that are taken up by cancer cells for synthesizing membranes. Skeletal muscles undergo proteolysis and secrete amino acids in response to pro-inflammatory cytokines released by cancer cells

Although mitochondria face substantial challenges in tumor cells and normal metabolic processes are deregulated, tumors retain an astounding capacity to perform oxidative phosphorylation. This is partially due to surrounding tissues fueling the tumor tissue [73]. This process was proposed through a two-compartment model in 2012 [74–76]. It is believed that glycolytic stromal cells produce both ketone bodies and l-lactate, which are used by oxidative epithelial cancer cells [77] (Fig. 3). These fibroblasts and adipocytes produce metabolites, which are then consumed by cancer cells to fuel cancer cell proliferation (see Fig. 3). Glycolytic cancer cells release lactate, which diffuses along its concentration gradient from the tumor towards blood vessels. Blood vessels eventually clear lactate [78]. In clinical tumors, the lactate amounts to 10–40 mM [79]. In addition to lactate, tumor cells also produce and secrete ammonia [80]. Lactate and ammonia secreted by some cancer cells can be taken up and utilized by other cancer cells in the same milieu [80]. In contrast to glycolytic cancer cells, oxidative cancer cells prefer lactate as the fuel source in comparison to glucose [81]. The oxidative cancer cells utilize lactate for respiration and produce ROS as a result. ROS with relatively longer half-life, hydrogen peroxide, can enter the membrane of fibroblasts in the tumor microenvironment. This causes oxidative damage resulting in a metabolic switch from oxidative metabolism to glycolytic metabolism in fibroblasts. These glycolytic fibroblasts release lactate and ketone bodies, which are in turn taken up by the oxidative tumor cells as fuel [80]. Fibroblasts also undergo autophagy to supply tumor cells with glutamine [80].

In addition to fibroblasts, skeletal muscles also supply several amino acids to tumor cells [82]. This process occurs when cancer cells secrete several pro-inflammatory cytokines such as TNF-α, IL-6, and IL-1 which causes oxidative stress and cachexia in skeletal muscles [83, 84]. As a result, skeletal muscles release amino acids by proteolysis [82]. Adipocytes in the vicinity of the tumor release fatty acids that serve as fuel for cancer cells [78, 82]. Pyruvate kinase isoforms have been implicated in mediation of this process, and glutamine appears to assist in this process [85–87]. The relationship between stromal cells and tumor cells has been referred to as micro-level commensalism [74]. Interestingly, in locally advanced NSCLC patients who received curative intent chemoradiotherapy (CRT), oral glutamine (Gln) supplementation significantly reduces grade 2–3 esophagitis and weight loss, with no negative impact on tumor control and survival outcomes [88]. It is worth noting that patients undergoing radiotherapy or chemotherapy have low plasma levels of Gln [89]. This would cause host cachexia, which is a severe problem in cancer patients [90, 91]. Thus, Gln supplementation would help patients undergoing CRT. However, for patients developing lung cancer or without cachexia, Gln supplementation would help cancer cells without benefiting the host.

## Mitochondria and OXPHOS play a pivotal role in drug resistance

Drug resistance is one of the major hurdles of an effective cancer therapy [92]. Numerous studies have shown that tumors that regress considerably in response to targeted therapy relapse as a more aggressive drug resistant form. Some populations of the cells remain dormant during therapy and subsequently become resistant. The resistant cells continue to establish their clonal populations, metastasis, and result in poor survival outcomes [92]. Therefore, tackling drug resistance is very vital to improve the treatment outcome.

Mitochondria have long been associated with ATP generation and ROS production in cancer. In this section we are delving into much lesser known roles of mitochondria in cancer cell survival and drug resistance. Recent studies have shown that several cancers have adapted and rewired their cells to rely more on mitochondrial OXPHOS for drug resistance [93–98]. Cancer cells have shifted gears to elevated OXPHOS through several different mechanisms ranging from gene upregulations to ectopic protein expressions [93, 99].

Cancer stem-like cells (CSCs) are associated with metastasis and resistance to adjuvant chemotherapy and radiotherapy [93]. Triple negative breast cancer (TNBC) exhibiting markers of CSCs are associated with poor outcomes. A recent study has shown that proto-oncogene MYC (a human gene over-expressed in various cancers that is homologous to an oncogene carried by the avian myelocytomatosis virus) and MCL1 (myeloid cell leukemia-1) protein (stimulates mitochondrial respiration when localized in mitochondrial matrix) are enhanced in CSCs of TNBC [93]. Both MYC and MCL1 promote OXPHOS. Elevated OXPHOS induced ROS which in turn activated HIF-1α, thereby conferring resistance to adjuvant chemotherapy [93, 95]. Similar observation was made in small cell lung cancer (SCLC) where CSCs exhibit elevated OXPHOS and preferential dependence on OXPHOS over glycolysis for energy. Oligomycin, an inhibitor of OXPHOS, abolished the tumor initiating abilities of CSCs, thereby implicating the role of OXPHOS in initiation of SCLC [100]. Similarly, NSCLC cells that are resistant to EGFR tyrosine kinase inhibitors, gefitinib and erlotinib, were shown to exhibit elevated OXPHOS accompanied by elevated glycolysis and activity in TCA cycle [99]. This metabolic shift to increased OXPHOS was found to be a result of MET (mesenchymal–epithelial transition factor) proto-oncogene expression in the mitochondrial membrane in addition to plasma membrane. Pharmacological inhibition of MET resulted in cytotoxicity and apoptosis [99]. Interestingly, cancer cells rewire metabolism by altering the localization of proteins and expressing them ectopically in mitochondria [93, 99]. 3-Oxoacid CoA-transferase 1 (OXAT1) and acetyl-CoA acetyltransferase 1 (ACAT1) are proteins localized in mitochondria and are involved in utilization of ketone bodies to aid in tumor growth and metastasis. Epithelial cancers like breast cancer overexpress these mitochondrial proteins to utilize ketone bodies like 3-hydroxybutyrate and aceto-acetate for fuel to promote tumor progression and metastasis in lungs [93, 99]. It has been shown by in silico drug designing and mammosphere assay that targeting the mitochondrial proteins—OXAT1 and ACAT1—can effectively inhibit activity and propagation of CSCs in breast cancer [101].

Activating mutations in KRAS oncogene is prevalent in lung, colon, and pancreatic cancers. A recent study that performed a CRISPR/Cas9 screening, identified several mitochondrial genes involved in ribosomes and translation shared lethal interactions with K-ras gene [102]. In renal cell carcinoma (RCC), an NADPH oxidase isoform, NOX4, localizes to the inner mitochondrial membrane, and subcellular redistribution of ATP levels from the mitochondria activates NOX4. NOX4-derived ROS inhibits P300/CBP-associated factor (PCAF)-dependent acetylation and lysosomal degradation of the pyruvate kinase-M2 isoform (PKM2). Silencing NOX4 sensitizes cultured and ex vivo freshly isolated RCC cells to etoposide-induced cell death in xenograft models and ex vivo cultures by acting via PKM2 [103].

A recent study showed that cisplatin-resistant lung adenocarcinoma cells exhibit higher mitochondrial membrane potential (MMP) and intracellular ATP levels than the non-resistant cells which confer migratory and invasive abilities to these cells [8]. Inhibition of mitochondrial complex I abolished the ability of the resistant cells to invade [8], suggesting the pivotal role of mitochondria in metastasis of resistant cells. Another recent study showed that mitochondrial oxygen consumption is a vital source of energy in paclitaxel-resistant lung adenocarcinoma cells [94]. In lung adenocarcinoma cell line A549, mitochondrial membrane depolarization increased during the initial phase where cells died in response to paclitaxel. However, as the cells gained resistance, normal mitochondrial membrane are restored by increased activity of catalase and glutathione peroxidase which counteracted the effect of increased ROS produced as result of paclitaxel treatment. Further, paclitaxel-resistant cells exhibited elevated extracellular acidification rates and oxygen consumption [94]. Several studies have shown that many cancer cells which are resistant, rely on mitochondrial oxidative phosphorylation for energy [96–98]. For example, cytarabine-resistant cells from PDX models of leukemia exhibited higher mitochondrial mass and consequently higher OXPHOS and ATP production [104].

Besides elevation of oxidative phosphorylation and oxygen consumption, several recent studies have shown that genes involved in mitochondrial biogenesis, mitochondrial electron transport chain, mitochondrial transcription factors as well as mitochondrial fission and fusion mediators are upregulated in resistant cancer cells [96–98, 105]. There is evidence that signaling axis involving TFAM—a transcriptional factor involved in mitochondrial biogenesis—is involved in conferring resistance to MAP Kinase inhibitors in melanoma. Additionally, cancer stem cells that are implicated in tumor progression and drug-resistance also exhibit elevated OXPHOS and depend on OXPHOS for energy. These studies showed that regardless of the type of cancer or method of interception, mitochondria play indispensable and multi-faceted roles in resistant cancer cells. Hence, targeting mitochondria could be an effective strategy to suppress drug-resistant cancer cells. Recent studies showed that targeted drug therapies, such as MAPK inhibitors, and first-line chemotherapeutic agents, such as platinum-based drugs, increase mitochondrial activity and OXPHOS [94, 105]. These studies showed that a combination of first-line therapeutic agents and mitochondrial targeting agents would plausibly serve as an effective strategy to curtail tumor progression.

Further, in silico analyses have shown that mitochondrial biogenesis could be used as a reliable factor for predicting outcomes in lung cancer patients [7]. In one study, about 33 mitochondrial-related genes were shown to correlate with NSCLC patient survival [77]. Another recent study showed difference in mitochondrial phenotypes between tumors that depend on glycolysis versus tumors that depend on OXPHOS [106]. Studies are underway to utilize mitochondrial imaging techniques to assess possible modes of prognosis and outcome for a patient [106]. Mitochondria can be specifically targeted via mitochondrial sirtuins that play an important role in cellular homeostasis and is a major regulator of metabolism in cancer cells. Therefore, it is a potential drug target and further studies are required to assess the ability of targeting mitochondrial sirtuins to specifically target cancer cells [107]. Apart from drugs that particularly target mitochondria, there are several vehicles like PEG coated CNTABT737 nanoparticles that are internalized into early endosomes via micropinocytosis and clathrin-mediated endocytosis and subsequently delivered into mitochondria [108].

Importantly, a recent study from the authors’ lab [109] indicated a link between heme and drug resistance in NSCLC tumor cells. Treatment of subcutaneous xenografts of NSCLC cells with a vascular disrupting agent, combretastatin A4-phosphate (currently in phase II clinical trials for non-squamous NSCLC), results in initial tumor regression followed by relapse. The treatment results in central necrosis; however, cells that constituted the rim of the tumor can proliferate to repopulate the tumor. These resistant cells exhibit increased levels of protein and enzymes involved heme synthesis and heme uptake, as well as elevated levels of oxygen-utilizing hemoproteins and mitochondrial respiratory chain complex subunits [109].

## Conclusions

Clearly, many lines of experimental evidence have convincingly demonstrated the importance of mitochondrial oxidative phosphorylation. As a key signaling and structural molecule for processes involved in oxygen utilization and oxidative metabolism, heme can impact lung tumorigenesis in a multi-faceted manner. It is worth noting that the *K*_*m*_ of heme synthetic enzymes and cytochrome c oxidase for oxygen is very low (< 1 µM or ~ 0.1%) [110–115]. This is below oxygen levels experienced by human cancer cells under hypoxia (0.3–4.2% oxygen saturation) [116]. Thus, both heme synthesis and mitochondrial respiration can be maintained under clinically defined tumor hypoxia. Therefore, the reliance of aggressive or drug-resistant tumor cells on OXPHOS does not conflict with the fact that aggressive and drug-resistant tumors are hypoxic.

## Abbreviations

ACAT1: acetyl-CoA acetyltransferase 1
ACLY: ATP citrate lyase
ALAS1: 5-aminolevulic acid synthase
ALK: anaplastic lymphoma kinase
ATP: adenosine triphosphate
CoA: coenzyme A
CRT: chemoradiotherapy
CSCs: cancer stem-like cells
CYP1B1: cytochrome P450 family 1 subfamily B member 1
EGFR: epidermal growth factor receptor
GAPDH: glyceraldehyde-3-phosphate dehydrogenase
Gln: glutamine
GTP: guanosine triphosphate
HCP1: heme carrier protein 1
HRG1: heme related gene 1
KRAS: kirsten rat sarcoma viral oncogene
LDH: lactate dehydrogenase
MCL1: myeloid cell leukemia-1
ME: malic enzyme
MET: mesenchymal–epithelial transition factor
MMP: mitochondrial membrane potential
MPC: mitochondrial pyruvate carrier
NAD: nicotinamide adenine dinucleotide
NADPH: nicotinamide adenine dinucleotide phosphate (reduced form)
NSCLC: non-small cell lung cancer
OXAT1: 3-oxoacid CoA-transferase 1
OXPHOS: oxidative phosphorylation
PCAF: P300/CBP-associated factor
PEP: phosphoenol pyruvate
PGC-1α: peroxisome proliferator-activated receptor gamma coactivator 1 alpha
PKM2: pyruvate kinase-M2 isoform
RCC: renal cell carcinoma
ROS: reactive oxygen species
SCLC: small cell lung cancer
TCA: tricarboxylic acid cycle
TFAM: transcription factor A, mitochondria
TNBC: triple negative breast cancer
